# Multi-Quantum Dots-Embedded Silica-Encapsulated Nanoparticle-Based Lateral Flow Assay for Highly Sensitive Exosome Detection

**DOI:** 10.3390/nano11030768

**Published:** 2021-03-18

**Authors:** Hyung-Mo Kim, Chiwoo Oh, Jaehyun An, Seungki Baek, Sungje Bock, Jaehi Kim, Heung-Su Jung, Hobeom Song, Jung-Won Kim, Ahla Jo, Dong-Eun Kim, Won-Yeop Rho, Jin-Young Jang, Gi Jeong Cheon, Hyung-Jun Im, Bong-Hyun Jun

**Affiliations:** 1 Department of Bioscience and Biotechnology, Konkuk University, Seoul 05029, Korea; hmkim0109@konkuk.ac.kr (H.-M.K.); ghj4067@konkuk.ac.kr (J.A.); bsj4126@konkuk.ac.kr (S.B.); susia45@gmail.com (J.K.); iamara0421@konkuk.ac.kr (A.J.); kimde@konkuk.ac.kr (D.-E.K.); 2 Department of Applied Bioengineering, Graduate School of Convergence Science and Technology, Seoul National University, Seoul 16229, Korea; ohs3636@gmail.com (C.O.); bsks1994@gmail.com (S.B.); 3 ZEUS Co. Ltd., Hwaseong 18636, Korea; hsjung@globalzeus.com; 4 BioSquare Inc., Seongnam 13209, Korea; hbsong@bio-square.com (H.S.); jwkim@bio-square.com (J.-W.K.); 5 School of International Engineering and Science, Jeonbuk National University, Jeonju 54896, Korea; rho7272@jbnu.ac.kr; 6 Department of Surgery and Cancer Research Institute, Seoul National University College of Medicine, Seoul 03080, Korea; jangjy4@snu.ac.kr; 7 Department of Nuclear Medicine, Seoul National University College of Medicine, Seoul 03080, Korea; 8 Cancer Research Institute, Seoul National University, Seoul 03080, Korea; 9 Department of Molecular Medicine and Biopharmaceutical Sciences, Graduate School of Convergence Science and Technology, Seoul National University, Seoul 16229, Korea

**Keywords:** exosomes, quantitative detection, lateral flow assay, multi-quantum dots-embedded silica-encapsulated silica nanoparticle, test strip

## Abstract

Exosomes are attracting attention as new biomarkers for monitoring the diagnosis and prognosis of certain diseases. Colorimetric-based lateral-flow assays have been previously used to detect exosomes, but these have the disadvantage of a high limit of detection. Here, we introduce a new technique to improve exosome detection. In our approach, highly bright multi-quantum dots embedded in silica-encapsulated nanoparticles (M–QD–SNs), which have uniform size and are brighter than single quantum dots, were applied to the lateral flow immunoassay method to sensitively detect exosomes. Anti-CD63 antibodies were introduced on the surface of the M–QD–SNs, and a lateral flow immunoassay with the M–QD–SNs was conducted to detect human foreskin fibroblast (HFF) exosomes. Exosome samples included a wide range of concentrations from 100 to 1000 exosomes/µL, and the detection limit of our newly designed system was 117.94 exosome/μL, which was 11 times lower than the previously reported limits. Additionally, exosomes were selectively detected relative to the negative controls, liposomes, and newborn calf serum, confirming that this method prevented non-specific binding. Thus, our study demonstrates that highly sensitive and quantitative exosome detection can be conducted quickly and accurately by using lateral immunochromatographic analysis with M–QD–SNs.

## 1. Introduction

Exosomes are small extracellular vesicles that contain various cellular products including proteins, lipids, mRNAs, and microRNAs, which can all exhibit cancer specific identities during oncogenesis. Exosomes can be obtained non-invasively from body fluids such as blood, saliva, and urine [1,2,3,4]. Due to these features, exosomes can provide a more convenient and non-invasive diagnostic method for cancer identification than tissue biopsies, which require surgery [5]. Many recent studies have used these advantages of the exosome to diagnose various diseases [6,7,8,9], and the field of exosome-based diagnostics is rapidly growing. However, the applications of exosome-based protein biomarkers have two major hurdles including the lack of a standardized exosome isolation method and suboptimal detection sensitivity. Thus far, the enzyme-linked immunosorbent assay (ELISA) is most widely used to detect and quantify exosomes with a high limit of detection (LOD). However, the long detection time of this method is disadvantageous as it requires expert technicians and many pretreatments [10,11].

The lateral flow assay (LFA) is one of the most affordable and rapid methods for target protein detection [12,13,14]. Relative to other methods, LFA is advantageous for clinical diagnostics because it does not require skilled technicians and results can be obtained quickly [15]. However, commercialized colorimetric LFAs have limited protein detection sensitivity and quantifiability [16,17].

Detection of target proteins by the fluorescent lateral flow assay (fLFA), based on fluorescent nanoparticles (NPs), has been proposed as an alternative to conventional colorimetric methods. Amongst the currently available fluorescent nanoparticles, quantum dots (QDots) have excellent optical and electronic properties. QDots are suitable for fLFA due to their high fluorescence intensity, narrow emission wavelength, high quantum yield (QY), and excellent chemical stability [18,19,20]. To apply QDots to fLFA, surface modifications are necessary to enhance biostability and promote antibody (Ab) conjugation on the nanoparticle surface. However, surface modifications usually cause decreased fluorescence intensity and lower QYs. We previously developed a large number of QDots (Multi-QDot) embedded in silica-encapsulated silica nanoparticles (M–QD–SNs) to overcome these problems [21,22]. Silica nanoparticles (SNs) with multiple QDots have several advantages including stronger fluorescence intensity than that of conventional single QD–SNs, the QY is not significantly reduced by the silica coating, and surface modification is simple [23].

In this study, M–QD–SNs were applied to fLFA to sensitively detect exosomes and perform quantitative fluorescence analysis to quickly and quantitatively measure exosome abundance.

## 2. Materials and Methods

### 2.1. Chemical and Materials

CdSeZnS/ZnS QDots (λem. 620 nm, surface capping with oleic acid) were purchased from Global ZEUS (Osan, Korea). Tetraethyl orthosilicate (TEOS), 3-mercaptopropyl trimethoxysilane (MPTS), (3-Aminopropyl) triethoxysilane (APTS), succinic anhydride, *N*,*N*-diisopropylethylamine (DIEA), *N*-(3-dimethylaminopropyl)-*N*′-ethylcarbodiimide (EDC) hydrochloride, *N*-hydroxysulfosuccinimide (Sulfo-NHS) sodium salt, 2-(*N*-Morpholino)ethanesulfonic acid hydrate (MES hydrate), phosphate-buffered saline (PBS, pH 7.4), TWEEN^®^ 20, and ethanolamine were purchased from Sigma Aldrich (St. Louis, MO, USA). Amino Polyethylene glycol (PEG) Acid (NH_2_–PEG–COOH, MW = 600 Da) was from Nanocs Inc. (New York, NY, USA). Dichloromethane (MC) was purchased from Samchun (Pyeongtaek, Korea). Ethyl alcohol (EtOH, 99.9%), aqueous ammonium hydroxide (NH_4_OH, 27%), and 1-methyl-2-pyrrolidinone (NMP) were purchased from Daejung (Siheung, Korea). The backing card, nitrocellulose (NC) membrane, absorbent pad, cassette, and monoclonal anti-rabbit IgG antibody were purchased from Bore Da Biotech Co. Ltd. (Seongnam, Korea). An anti-CD63 antibody (CD63 Ab) was purchased from Santa Cruz Biotechnology (Dallas, TX, USA). Depleted fetal bovine serum was purchased from System Biosciences (Palo Alto, CA, USA). Total exosome isolation reagent and a size marker were purchased from Thermo Fisher (Waltham, MA, USA). The primary antibodies, CD63 and CD81 Ab, were purchased from Santa Cruz Biotechnology (Dallas, TX, USA). Goat anti-mouse IgG Ab was purchased from Invitrogen (Carlsbad, CA, USA).

### 2.2. Synthesis of Silica Coated Multi-Quantum Dot (M–QD–SNs)

Silica NPs were synthesized by a modified Stöber method [24]. TEOS (1.6 mL), NH_4_OH (3 mL), and absolute EtOH (40 mL) were stirred for 20 h at 25 °C. The mixture was washed several times with absolute EtOH by centrifugation at 8500 rpm for 10 min. Then, silica NPs (200 mg) were mixed with absolute EtOH (4 mL), MPTS (200 μL), and NH_4_OH (40 μL) for 12 h at 25 °C. The mixture was washed several times with absolute EtOH by centrifugation at 8500 rpm for 10 min.

To introduce QDots to the surface of thiol-modified silica NPs, silica NPs (10 mg) were mixed with MC (4 mL) and QDots (7 mg), and the mixture was incubated in a shaking incubator for 3 h at 25 °C. The mixture was then added to MPTS (50 μL) and NH_4_OH (50 μL), accompanied by vigorous stirring for 3 h at 25 °C. The mixture was washed several times with absolute EtOH by centrifuging at 8500 rpm for 10 min. For silica coating, washed NPs were dispersed in absolute EtOH (5 mL) and added to TEOS (50 μL) and NH_4_OH (50 μL) by vigorously stirring for 20 h at 25 °C. The final products (M–QD–SNs) were washed several times with absolute EtOH by centrifugation at 8500 rpm for 10 min and re-dispersed in absolute EtOH.

### 2.3. M–QD–SNs–Antibody Conjugates

To introduce amine groups to the surface, M–QD–SNs (1 mg) were mixed with APTS (10 μL) and NH_4_OH (10 μL) for 1 h at 25 °C. The mixture was washed several times with NMP by centrifugation at 13,000 rpm for 10 min. To introduce carboxyl groups onto the surface of amine-functionalized M–QD–SNs, M–QD–SNs were dispersed in NMP (500 μL consisting of 1.75 mg of succinic anhydride), and the mixture was added to DIEA (3.05 μL) and stirred for 2 h at 25 °C. The mixture was washed several times with deionized water (DW) by centrifugation at 13,000 rpm for 10 min, followed by re-dispersion in MES hydrate (50 mM). Carboxyl-functionalized M–QD–SNs were added to EDC (2 mg) and sulfo-NHS (2 mg), and the mixture was stirred for 30 min at 25 °C. The supernatant was removed, followed by dispersion in MES hydrate (50 mM). The activation group-functionalized M–QD–SNs were added to NH_2_–PEG–COOH (1.6 mM), and the mixture was stirred for 2 h at 25 °C. To deactivate the activated group, M–QD–SNs–PEG–COOH were added to ethanolamine (3.2 μL) and stirred for 30 min at 25 °C. The mixture was washed several times with DW by centrifugation at 13,000 rpm for 10 min and was re-dispersed in MES hydrate (50 mM). M–QD–SNs–PEG-COOH was added to EDC (2 mg) and sulfo-NHS (2 mg), and the mixture was stirred for 30 min at 25 °C. The supernatant was removed, and the pellet was dispersed in MES hydrate (50 mM). The activation group-supplemented M–QD–SNs–PEG-COOH was added to CD63 Ab, and the mixture was stirred for 2 h at 25 °C. After centrifugation, M–QD–SNs–PEG-CD63 Ab was added to ethanolamine (3.2 μL) and the mixture was stirred for 30 min at 25 °C. The mixture was washed several times with 0.5% bovine serum albumin (BSA) by centrifugation at 13,000 rpm for 10 min, and it was re-dispersed in 0.5% BSA.

### 2.4. Test Strip Preparation

The test strip consisted of a backing card, NC membrane, and absorbent pad. After assembling the NC membrane on the backing card, the test line was sprayed with CD63 Ab (1 mg/mL) using a dispenser, and the control line was sprayed with monoclonal rabbit IgG Ab (10 mg/mL). The assembly was dried for approximately 2 h. Then, 0.1% BSA was applied to the assembly and dried for at least a day. Finally, the absorbent pad was assembled on the backing card. After cutting to 6 mm, the test strip was completed.

### 2.5. Fluorescence Assay Procedure

A mixture of 30 μL of human foreskin fibroblast (HFF) exosomes diluted by using 0.5% PBST (pH 7.4) and 3 µL of M–QD-SNs-CD63 Ab (0.3 μg) that considered concentration with specifications of test strip reader was combined in one well of a 96-well plate. Immediately after, the test strip was then placed in the well and the mixture was allowed to spread for 10 min. After completion of the reaction, the strip (in a cassette) was analyzed using a test strip reader (ESEQuant LR3 FL Premium Reader; Qiagen). In addition, the LOD based on the photoluminescence (PL) intensity obtained at each concentration (0 and 100–1000 exosomes/µL) was calculated by the following equation.

$$y=6276.17+\frac{14.99-6276.17}{1+{\left(\frac{x}{1444.11}\right)}^{2.23}}$$

### 2.6. Human Foreskin Fibroblast (HFF) Exosome Extraction

The HFF-1 cell lines were obtained from Department of Molecular Medicine and Biopharmaceutical Sciences, Graduate School of Convergence Science and Technology, Seoul National University. The HFF-1 cell lines were incubated at 37 °C. Dulbecco’s modified Eagle’s medium (DMEM) was exchanged with extracellular vesicle (EV)-depleted fetal bovine serum before HFF exosomes were extracted. The following day, the medium with HFF cells was centrifuged at 3000 rpm for 30 min at 4 °C to separate apoptotic cells. The supernatant liquid was mixed with DMEM and a total exosome isolation reagent. The mixed liquid was maintained overnight at 4 °C. The following day, the mixed liquid was centrifugated at 10,000× *g* for 60 min at 4 °C. The pellet was resuspended in PBS. Resuspended HFF exosomes were stored at −20 °C.

### 2.7. Control Liposome Preparation

The self-assembly method was used to make a control liposome with 1,2-distearoyl-sn-glycero-3-phosphocholine (DSPC), 1,2-distearoyl-sn-glycero-3-phosphoethanolamine-*N*-[methyl (polyethylene glycol)-5000] (DSPE-PEG (5000)-CH_3_), and cholesterol. DSPC, DSPE-PEG (5000)-CH_3_, and cholesterol were mixed in a 1:1:1 molar ratio in chloroform. The mixed chloroform solution was evaporated and the lipid layer that formed was vacuumed overnight. The completely removed lipid layer was turned into multilamellar vesicles (MLVs) by sonication. The MLV solution was formed into the desired liposome of 100 nm by ultrasonication and filtration with a 0.2 µm pore syringe filter.

### 2.8. Exosome Immunoblotting

HFF exosomes were degraded using a heat block for 3 min at 97 °C. Gel electrophoresis was performed using a size marker and the prepared sample at 120 V for 120 min. The sample was transferred to a membrane at 350 mA for 60 min at 4 °C. The transferred membrane was soaked in skimmed milk for 60 min to prevent non-specific binding. After incubation at 4 °C overnight with the primary antibody, CD63 or CD81 Ab in 5% BSA, a secondary antibody, goat anti-mouse IgG Ab in 5% BSA, was incubated with the membrane. The exosome immunoblot images were then acquired.

### 2.9. Characterization

Transmission electron microscopy images of NPs and HFF exosomes were obtained using a JEM-2010 system (JEOL, Tokyo, Japan). Cryo-transmission electron microscopy images of the control liposome were obtained using a Talos L120C (Thermo Fisher, Waltham, MA, USA). The UV/Vis absorbance of the NPs was measured using a UV/Vis spectrophotometer (Mecasys OPTIZEN POP, Daejeon, Korea). The fluorescence emission spectrum was obtained using a Model Cary Eclipse (Agilent Technologies, Santa Clara, CA, USA). The concentration of the extracted exosome was calculated through a colorimetric assay (23227; Thermo Fisher, Waltham, MA, USA) at 562 nm. The size and quantity of the calculated exosome were measured using NanoSight LM20 (Amesbury, Wiltshire, UK). Particles with a size of 30 to 150 nm were captured for 20 s at 30 frames. The number of exosomes was calculated using the movement of the captured exosome with the NTA software (version 3.1; NanoSight Ltd., Salisbury, England). The size of M–QD–SNs was measured using the NanoSight LM20. Exosome sizes were measured using a dynamic light scattering spectrophotometer (DLS-7000; Otsuka Electronics, Osaka, Japan). Zeta potential was measured using the DLS. QY was measured using QE-2000 (Otsuka Electronics). The photoluminescent intensity of the developed strips was measured using LR3 (QIAGEN, Hilden, Germany). Test strips and exosome immunoblot images were analyzed using an image analyzer (LAS-4000; Fujifilm, Tokyo, Japan).

## 3. Results

### 3.1. Synthesis and Characterization of M–QD–SNs

**Figure 1:**
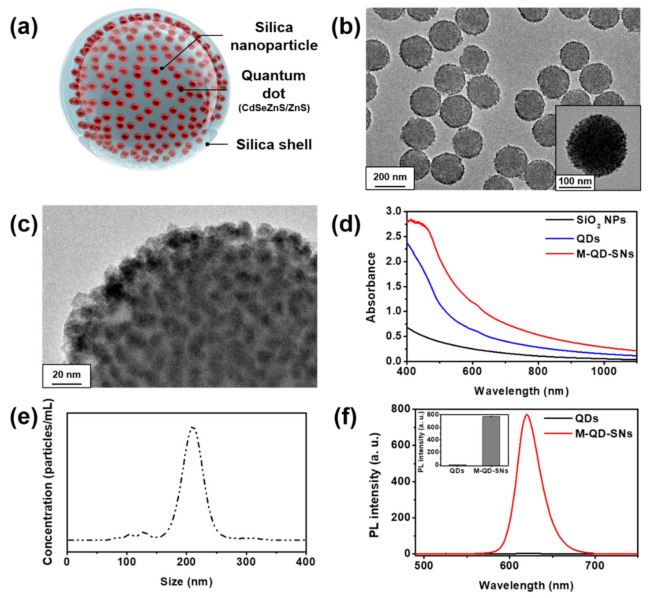
Characterization of silica coated multi-quantum dot (M–QD–SNs). (**a**) Schematic representation, and (**b**) transmission electron microscopy (TEM) image of M–QD–SNs. (**c**) Surface of M–QD–SNs. (**d**) UV/vis extinction spectra of silica NPs, QDots, and M–QD–SNs. (**e**) Size distribution of M–QD–SNs. (**f**) Comparison of PL intensity at 532 nm excitation wavelength between single QDots and M–QD–SNs (inset: comparison of max intensity).

For sensitive, rapid, and quantitative detection of exosomes, highly bright fluorescent M–QD–SNs (Figure 1a) were applied to the fLFA method. First, we synthesized M–QD–SNs through the modification of previously reported methods [21,25]. Briefly, we synthesized silica nanoparticles (silica NPs) of approximately 160 nm in size by ligand exchange and a sol–gel process based on the modified Stöber method, followed by the introduction of a thiol group on the surface for QDots embedding [24]. Then, abundant QDots were introduced onto the manufactured silica nanoparticles. This step differed from the previous method as we used CdSeZnS/ZnS QDots with an alloyed structure because alloy-type CdSeZnS/ZnS QDots are more stable after surface modification than CdSeZnS/ZnS QDots of the type-1 core/shell configuration, and they exhibited high QY than the type-1 structured QDot because of high photostability [26,27,28]. The QDot introduced silica NPs were then coated with a silica shell to prevent aggregation and to easily introduce various functional groups onto the surface of the particles [29].

The prepared M–QD–SNs were not aggregated and multiple QDots were embedded on the surface of SNs (Figure 1b). The silica shell successfully coated the surface of clustered QDots on the SNs (Figure 1c). Absorbance comparisons between M–QD–SNs, silica NPs, and QDots showed that the UV/Vis spectra was similar between M–QD–SNs and QDots (Figure 1d). Furthermore, the result of the UV/Vis spectrum indicated that QDots were successfully embedded onto the surface of the silica NPs. The newly synthesized M–QD–SNs also had a uniform size distribution (224 ± 2 nm, Figure 1e), as determined using a nanoparticle tracking analyzer. Additionally, the fluorescence signals were stronger, and the maximum fluorescence signals (at 620 nm) were approximately 300 times higher for the M–QD–SNs than for single hydrophilic QDots (Figure 1f).

These results indicated that M–QD–SNs were sufficient for fLFA using test strips with high sensitivity, consistent with the results of a previous study [21]. The QYs of single hydrophobic QDots and M–QD-SNs were 96% and 61%, respectively, and M–QD-SNs maintained more than 60% QY compared to the existing single hydrophobic QDot (Figure S1). This decrease in QY was caused not only during the ligand exchange step for conjugation of QDots on the silica core, but was also caused by silica core and silica shells, which can inhibit the absorption of external light and irradiation of fluorescence. Although a decrease of QY was seen, this result was consistent with previous results where the single M–QD–SN was brighter than the single QDot [21,25].

### 3.2. Characterization of Extracted HFF Exosomes and Conjugation of CD63 Ab to M–QD–SNs

**Figure 2:**
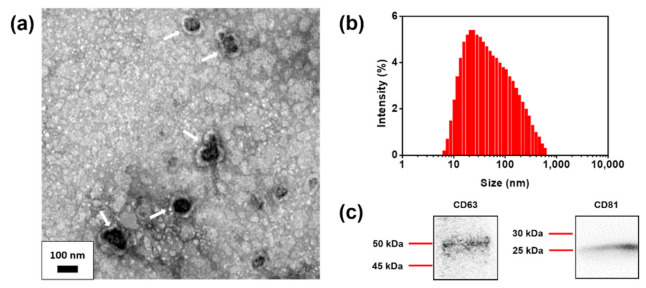
Characterization of human foreskin fibroblast (HFF) exosomes. (**a**) Transmission electron microscopy (TEM) image of HFF exosomes, indicated by arrows. (**b**) Size distribution of HFF exosomes using dynamic light scattering (DLS). (**c**) HFF exosomes immunoblotting using exosome marker proteins, CD63 and CD81.

We selected HFF exosomes as a study model. Extracted HFF exosomes were found to be spherical in shape by transmission electron microscopy (TEM) (Figure 2a). Dynamic light scattering (DLS, ZETASIZER Nano ZS, Malvern Instrument Ltd., Malvern, UK) demonstrated that the size of isolated exosomes was 84.1 ± 105.3 nm, which is within the reported size range of exosomes (Figure 2b). HFF exosomes were extracted using the Total Exosome Isolation Reagent (4478359; Invitrogen, Carlsbad, CA, USA). Characterization by the immunoblot assay showed that HFF exosomes were highly enriched for the markers CD63 and CD81, which belong to the tetraspanin proteins that characterize exosomes (Figure 2c).

Before proceeding with the experiment, the surface of the M–QD–SNs was additionally modified by CD63 Ab conjugation as described in Figure S2. (sc-5275; Santa Cruz Biotechnology, Dallas, TX, USA). NH_2_-PEG-COOH was conjugated to prevent particle aggregation. The CD63 Ab was conjugated to the M–QD–SN surface, as confirmed by the higher zeta potential than that of the previous step, as in a previous study (Figure S3) [30].

### 3.3. Detection of HFF Exosomes at Various Concentration with M-QD-SNs for Fluorescent Lateral Flow Assay (fLFA)

**Figure 3:**
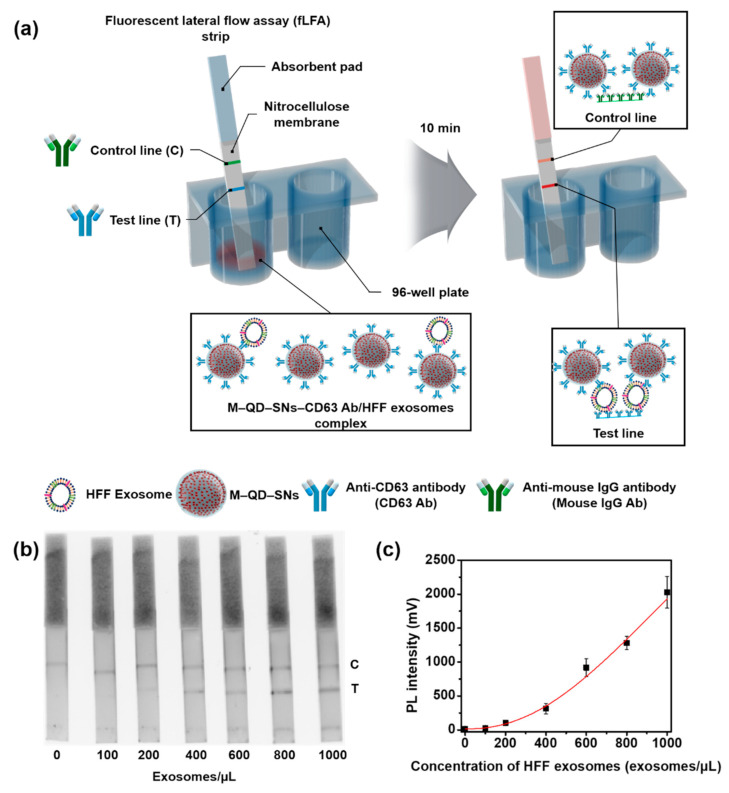
Detection of HFF exosomes using M–QD–SNs in the fLFA. (**a**) Schematic illustration of the configuration and measurement principle of the M–QD–SNs and exosome detection system. (**b**) Qualitative analysis of HFF exosomes by concentration (0, 100, 200, 400, 600, 800, and 1000 exosomes/µL) under LAS-4000. (**c**) Correlation analysis of the detectable concentration by a lateral flow immunoassay (PL intensity) and HFF exosome concentrations from 100 to 1000 exosomes/µL. Error bars denote the standard deviations of three independent experiments.

The overall scheme of HFF exosome detection by M–QD–SNs using a test strip is depicted in Figure 3a. After CD63 Ab-conjugated M-QD-SNs and HFF exosomes were combined in the well of a 96-well plate, test strips were inserted, and the mixture bound to the test line of the test strip. Unbound components of the mixture were combined with anti-mouse IgG Ab in the control line of the test strip for system validation.

We aimed to develop a fast and simple method for exosome detection that does not rely on large and complex instruments or skilled personnel, and which can be achieved using a small amount of sample and a strip reader for analysis (ESEQuant LR3 FL Premium Reader; Qiagen, Hilden, Germany). To analyze low exosome concentrations, the sensitivity of the test strip was observed using an image analysis system (LAS-4000; Fujifilm, Tokyo, Japan) [28]. The detection sensitivity for HFF exosomes was calibrated with standard HFF exosome samples at a concentration range of 0–1000 exosomes/µL diluted in 0.5% Tween 20-phosphate buffered saline (PBS, 0.5% PBST solution). As shown in Figure 3b, the fluorescence intensity depended on the concentration of HFF exosomes without non-specific binding in the test line of the test strip. Additionally, a calibration curve for HFF exosome quantification was obtained by recording the fluorescence intensity of the test line for different HFF exosome concentrations (Figure 3c). A polynomial model was fit to the data with R^2^ = 0.985. The LOD was 118 exosomes/µL, which was at least 11 times lower than previously reported values (Table 1). Thus, the bright fluorescence intensity of M–QD–SNs is highly advantageous for the highly sensitive detection of exosomes without non-specific binding.

### 3.4. Identification of HFF Exosome Detection Specificity by M-QD-SNs in fLFA

**Figure 4:**
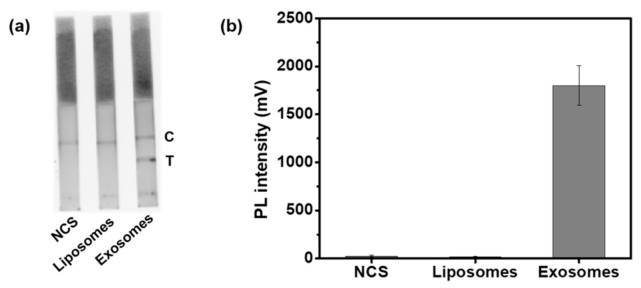
Characterization of the M–QD–SNs test strip system specificity. (**a**) Fluorescence image and corresponding (**b**) quantification of test strips with 33 μL of sample. Strips contained exosomes (at a concentration of 1000 exosomes/μL), standard liposomes (negative control), or newborn calf serum (NCS, blank control).

To determine the specificity of the system for HFF exosome detection, strips with liposomes or with newborn calf serum were used as negative controls. Liposomes, like exosomes, consist of a single lipid bilayer, and they lack proteins including CD63 on the surface, making them a suitable negative control [34]. Synthesized liposomes had a spherical shape (Figure S4a) and uniform size distribution with a hydrodynamic size of 97 ± 30 nm (Figure S4b). While the test line for HFF exosomes showed a fluorescent band, control strips did not have fluorescent bands at the test lines (Figure 4a). Using a strip reader for analyses, a remarkably high fluorescence value was obtained for the test line of HFF exosomes (Figure 4b). These results demonstrate that this system for HFF exosome detection using a test strip with M–QD–SNs achieved high specificity. This system could easily be applied for the detection of other types of exosomes.

## 4. Conclusions

In summary, our study demonstrates that HFF exosomes can be detected using CD63 Ab-conjugated M–QD–SNs in the fLFA with test strips. M–QD–SNs with multiple number of QDots on the surface represented high PL intensity without a significant decrease in QY compared with previous studies using existing hydrophobic QDots. Furthermore, the wide absorbance range and strong fluorescence signals were suitable for use with a commercial strip reader. TEM and DLS indicated that HFF exosomes maintained a uniform size. The fLFA with M-QD-SNs was able to detect a wide range of HFF exosomes from 1000 to 100 exosomes/uL, and the LOD was 117.94 exosomes/uL, which was approximately 11 times lower than LODs achieved by previous systems. Furthermore, non-specific binding was not observed when liposomes were used as a control, reflecting the high specificity of the system. Together, these results demonstrate that exosomes can be rapidly detected by the fLFA with M–QD–SNs, which is a highly sensitive and quantitative method.

## Figures and Tables

**Table 1:** Comparison of nanoparticle types and limit of detection (LOD) in test strip studies of exosome detection.

No.	Signal-Generating Reagent	LOD (exosomes/µL)	Reference
1	Gold nanoparticles	8.54 × 10^5^	[31]
2	Double gold–nanoparticle conjugates	1.3 × 10^3^	[32]
3	Au@Pd nanopopcorn	1.4 × 10^4^	[33]
4	M–QD–SNs	0.2 × 10^2^	This study

## Data Availability

The data presented in this study are available on request from the corresponding authors.

## Supplementary Materials

The following are available online at https://www.mdpi.com/2079-4991/11/3/768/s1, Figure S1: Comparison of the quantum yields of single QDots and M–QD–SNs; Figure S2: Schematic illustration of M-QD-SNs-CD63 Ab fabrication; Figure S3: Difference in zeta potential in each step of the preparation of M–QD–SNs conjugated with CD63 Ab; Figure S4: Characterization of liposomes (a) cryo-TEM image of liposomes, and (b) size distribution of control liposomes using DLS.

## References

[1] Kosaka N., Kogure A., Yamamoto T., Urabe F., Usuba W., Prieto-Vila M., Ochiya T. (2019). Exploiting the message from cancer: The diagnostic value of extracellular vesicles for clinical applications. Exp. Mol. Med..

[2] Huang T., Deng C.-X. (2019). Current Progresses of Exosomes as Cancer Diagnostic and Prognostic Biomarkers. Int. J. Biol. Sci..

[3] LeBleu V.S., Kalluri R. (2020). Exosomes as a Multicomponent Biomarker Platform in Cancer. Trends Cancer.

[4] Théry C., Zitvogel L., Amigorena S. (2002). Exosomes: Composition, biogenesis and function. Nat. Rev. Immunol..

[5] Soung Y.H., Ford S., Zhang V., Chung J. (2017). Exosomes in cancer diagnostics. Cancers.

[6] Khodashenas S., Khalili S., Moghadam M.F. (2019). A cell ELISA based method for exosome detection in diagnostic and therapeutic applications. Biol. Lett..

[7] Im H., Shao H., Park Y.I., Peterson V.M., Castro C.M., Weissleder R., Lee H. (2014). Label-free detection and molecular profiling of exosomes with a nano-plasmonic sensor. Nat. Biotechnol..

[8] Hikita T., Miyata M., Watanabe R., Oneyama C. (2018). Sensitive and rapid quantification of exosomes by fusing luciferase to exosome marker proteins. Sci. Rep..

[9] Jalalian S.H., Ramezani M., Jalalian S.A., Abnous K., Taghdisi S.M. (2019). Exosomes, new biomarkers in early cancer detection. Anal. Biochem..

[10] Sakamoto S., Putalun W., Vimolmangkang S., Phoolcharoen W., Shoyama Y., Tanaka H., Morimoto S. (2018). Enzyme-linked immunosorbent assay for the quantitative/qualitative analysis of plant secondary metabolites. J. Natl. Med..

[11] Jia C.-P., Zhong X.-Q., Hua B., Liu M.-Y., Jing F.-X., Lou X.-H., Yao S.-H., Xiang J.-Q., Jin Q.-H., Zhao J.-L. (2009). Nano-ELISA for highly sensitive protein detection. Biosens. Bioelectron..

[12] Lu T., Zhu K.-D., Huang C., Wen T., Jiao Y.-J., Zhu J., Zhang Q., Ding S.-N. (2020). Rapid detection of Shiga toxin type II using lateral flow immunochromatography test strips of colorimetry and fluorimetry. Analyst.

[13] Wyatt M.C., Beswick A.D., Kunutsor S.K., Wilson M.J., Whitehouse M.R., Blom A.W. (2016). The Alpha-Defensin Immunoassay and Leukocyte Esterase Colorimetric Strip Test for the Diagnosis of Periprosthetic Infection: A Systematic Review and Meta-Analysis. JBJS.

[14] Koczula K.M., Gallotta A. (2016). Lateral flow assays. Essays Biochem..

[15] Li S., Jin Q., Jiang X., Park J.J.J.H. (2013). Frontier and Future Development of Information Technology in Medicine and Education: ITME 2013.

[16] Yang H., Li D., He R., Guo Q., Wang K., Zhang X., Huang P., Cui D. (2010). A novel quantum dots–based point of care test for syphilis. Nanoscale Res. Lett..

[17] Eltzov E., Guttel S., Low Yuen Kei A., Sinawang P.D., Ionescu R.E., Marks R.S. (2015). Lateral flow immunoassays–from paper strip to smartphone technology. Electroanalysis.

[18] Berlina A.N., Taranova N.A., Zherdev A.V., Vengerov Y.Y., Dzantiev B.B. (2013). Quantum dot-based lateral flow immunoassay for detection of chloramphenicol in milk. Anal. Bioanal. Chem..

[19] Fu Y., Zhang J., Lakowicz J.R. (2009). Silver-enhanced fluorescence emission of single quantum dot nanocomposites. Chem. Commun..

[20] Wang J., Liu G., Wu H., Lin Y. (2008). Quantum-Dot-based electrochemical immunoassay for high-throughput screening of the prostatespecific antigen. Small.

[21] Jun B.H., Hwang D.W., Jung H.S., Jang J., Kim H., Kang H., Kang T., Kyeong S., Lee H., Jeong D.H. (2012). Ultrasensitive, Biocompatible, Quantum-Dot-Embedded Silica Nanoparticles for Bioimaging. Adv. Funct. Mater..

[22] Ha Y., Jung H.S., Jeong S., Kim H.M., Kim T.H., Cha M.G., Kang E.J., Pham X.H., Jeong D.H., Jun B.H. (2019). Fabrication of Remarkably Bright QD Densely-Embedded Silica Nanoparticle. Bull. Korean Chem. Soc..

[23] Goftman V.V., Markin A.V., De Saeger S., Goryacheva I.Y. Multicolored silica coated CdSe core/shell quantum dots. Proceedings of the Saratov Fall Meeting 2015: Third International Symposium on Optics and Biophotonics and Seventh Finnish-Russian Photonics and Laser Symposium (PALS).

[24] Stöber W., Fink A., Bohn E. (1968). Controlled growth of monodisperse silica spheres in the micron size range. J. Colloid Interface Sci..

[25] Jo A., Kim T.H., Kim D.-M., Kim H.-M., Seong B., Kim J., Pham X.-H., Jung H.S., Lee S.H., Jeong D.H. (2020). Sensitive detection of virus with broad dynamic range based on highly bright quantum dot-embedded nanoprobe and magnetic beads. J. Ind. Eng. Chem..

[26] Cho J., Jung Y.K., Lee J.-K., Jung H.-S. (2017). Highly efficient blue-emitting CdSe-derived core/shell gradient alloy quantum dots with improved photoluminescent quantum yield and enhanced photostability. Langmuir.

[27] Lee K.-H., Lee J.-H., Kang H.-D., Han C.-Y., Bae S.M., Lee Y., Hwang J.Y., Yang H. (2014). Highly fluorescence-stable blue CdZnS/ZnS quantum dots against degradable environmental conditions. J. Alloys Compd..

[28] Jun S., Jang E. (2013). Bright and stable alloy core/multishell quantum dots. Angew. Chem. Int. Ed..

[29] Wolcott A., Gerion D., Visconte M., Sun J., Schwartzberg A., Chen S., Zhang J.Z. (2006). Silica-coated CdTe quantum dots functionalized with thiols for bioconjugation to IgG proteins. J. Phys. Chem. B.

[30] Jiang W., Kim B.Y., Rutka J.T., Chan W.C. (2008). Nanoparticle-mediated cellular response is size-dependent. Nat. Nanotechnol..

[31] Oliveira-Rodríguez M., López-Cobo S., Reyburn H.T., Costa-García A., López-Martín S., Yáñez-Mó M., Cernuda-Morollón E., Paschen A., Valés-Gómez M., Blanco-López M.C. (2016). Development of a rapid lateral flow immunoassay test for detection of exosomes previously enriched from cell culture medium and body fluids. J. Extracell. Vesicles.

[32] Wu T., Yang Y., Cao Y., Huang Y., Xu L.-P., Zhang X., Wang S. (2018). Enhanced lateral flow assay with double conjugates for the detection of exosomes. Sci. China Chem..

[33] Cheng N., Song Y., Shi Q., Du D., Liu D., Luo Y., Xu W., Lin Y. (2019). Au@Pd nanopopcorn and aptamer nanoflower assisted lateral flow strip for thermal detection of exosomes. Anal. Chem..

[34] Antimisiaris S.G., Mourtas S., Marazioti A. (2018). Exosomes and Exosome-Inspired Vesicles for Targeted Drug Delivery. Pharmaceutics.

