# Supplementary material for: Multi-Quantum Dots-Embedded Silica-Encapsulated Nanoparticle-Based Lateral Flow Assay for Highly Sensitive Exosome Detection
Source: Nanomaterials (Basel). 2021 Mar 18;11(3):768. doi: 10.3390/nano11030768 (PMC8002883; doi:10.3390/nano11030768)
Supplement: Supplementary file 1 [file nanomaterials-11-00768-s001.pdf]

Multi-Quantum Dots-Embedded Silica-Encapsulated Nanoparticle-Based Lateral Flow Assay for Highly Sensitive Exosome Detection

Hyung-Mo Kim [1,†], Chiwoo Oh [2,†], Jaehyun An [1], Seungki Baek [2], Sungje Bock [1], Jaehi Kim [1], Heung-Su Jung [3], Hobeom Song [4], Jung-Won Kim [4], Ahla Jo [1], Dong-Eun Kim [1], Won-Yeop Rho [5], Jin-Young Jang [6], Gi Jeong Cheon [7,8,9,*], Hyung-Jun Im [2,9*] and Bong-Hyun Jun [1,*]

[1] Department of Bioscience and Biotechnology, Konkuk University, Seoul, 05029, Korea; hmkim0109@konkuk.ac.kr (H.-M.K.); ghj4067@konkuk.ac.kr (J.A.); bsj4126@konkuk.ac.kr (S.B.); susia45@gmail.com (J.K.); iamara0421@konkuk.ac.kr (A.J.); kimde@konkuk.ac.kr (D.-E.K.)

[2] Department of Applied Bioengineering, Graduate School of Convergence Science and Technology, Seoul National University, Seoul, 16229, Korea; ohs3636@gmail.com (C.O.); bsks1994@gmail.com (S.B.) iiihjjj@snu.ac.kr (H.-J.I.)

[3] ZEUS Co. Ltd., Hwaseong, 18636, Korea; hsjung@globalzeus.com

[4] BioSquare Inc., Seongnam, 13209, Korea; hbsong@bio-square.com (H.S.); jwkim@bio-square.com (J.-W.K.)

[5] School of International Engineering and Science, Jeonbuk National University, Jeonju, 54896, Korea; rho7272@jbnu.ac.kr

[6] Department of Surgery and Cancer Research Institute, Seoul National University College of Medicine, Seoul, 03080, Korea; jangjy4@snu.ac.kr

[7] Department of Nuclear Medicine, Seoul National University College of Medicine, Seoul, 03080, Korea

[8] Cancer research institute, Seoul National University, Seoul, 03080, Korea

[9] Department of Molecular Medicine and Biopharmaceutical Sciences, Graduate School of Convergence Science and Technology, Seoul National University, Seoul, 16229, Korea; iiihjjj@snu.ac.kr (H.-J.I.)

* Correspondence: larrycheon@snu.ac.kr (G.J.C.); iiihjjj@snu.ac.kr (H.-J.I.); bjun@konkuk.ac.kr (B.-H.J.); Tel.: +82-2-2072-3386 (G.J.C.); +82-31-888-9187 (H.-J.I.); +82-2-450-0521 (B.-H.J.)

† These authors contributed equally to this work.

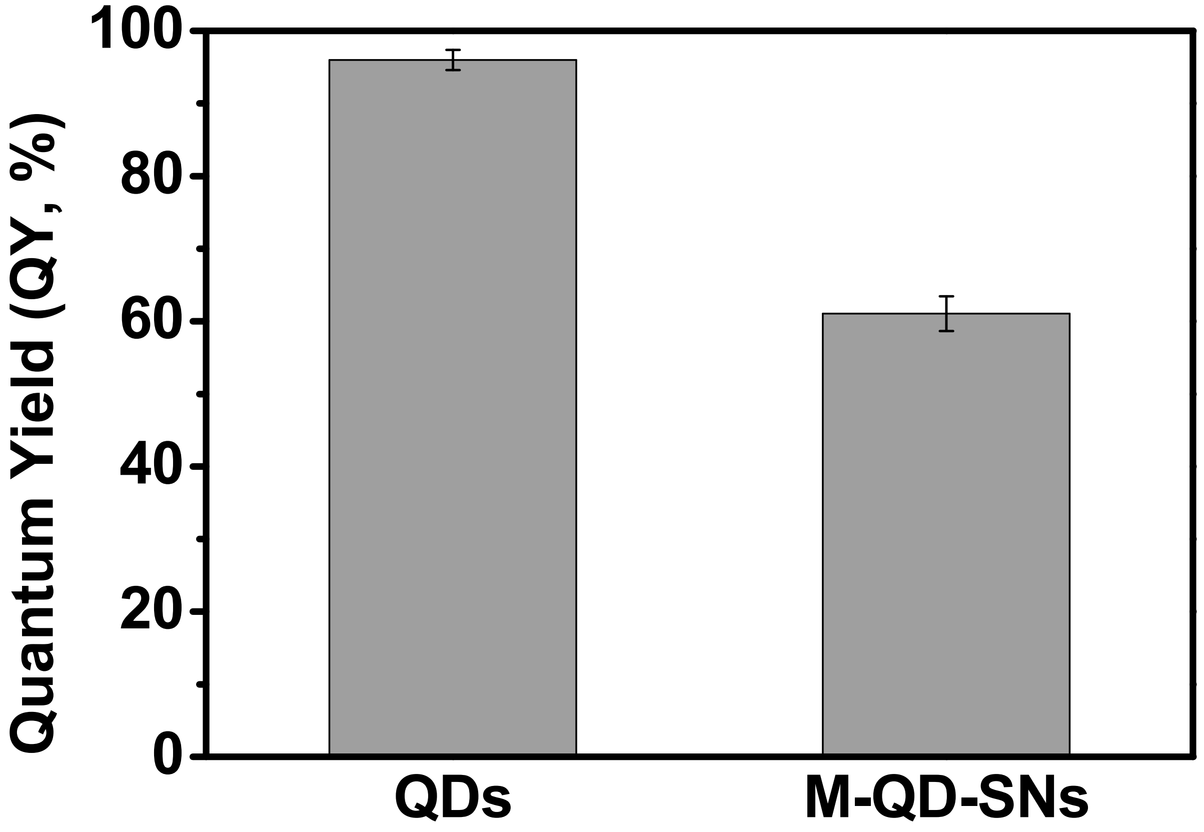

Figure S1. Comparison of the quantum yields of single QDots and M–QD–SNs.

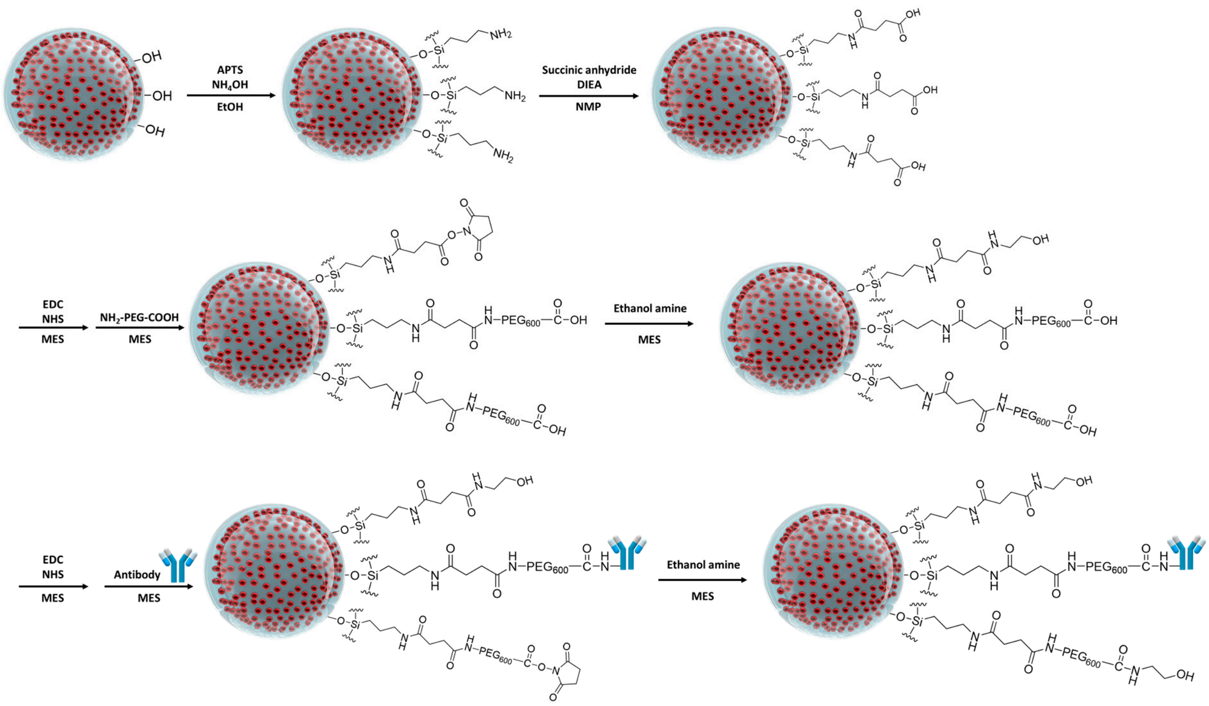

Figure S2. Schematic illustration of M–QD–SNs–CD63 Ab fabrication.

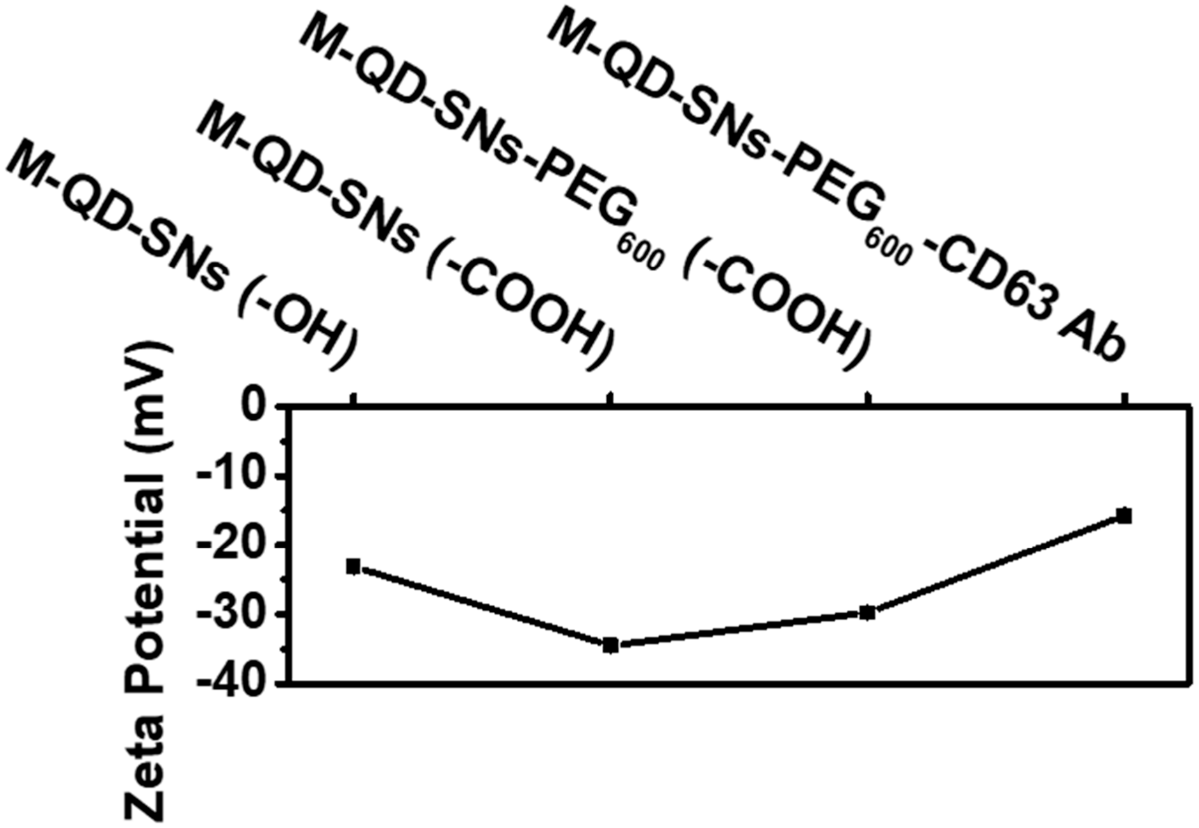

Figure S3. Difference in zeta potential in each step of the preparation of M–QD–SNs conjugated with CD63 Ab.

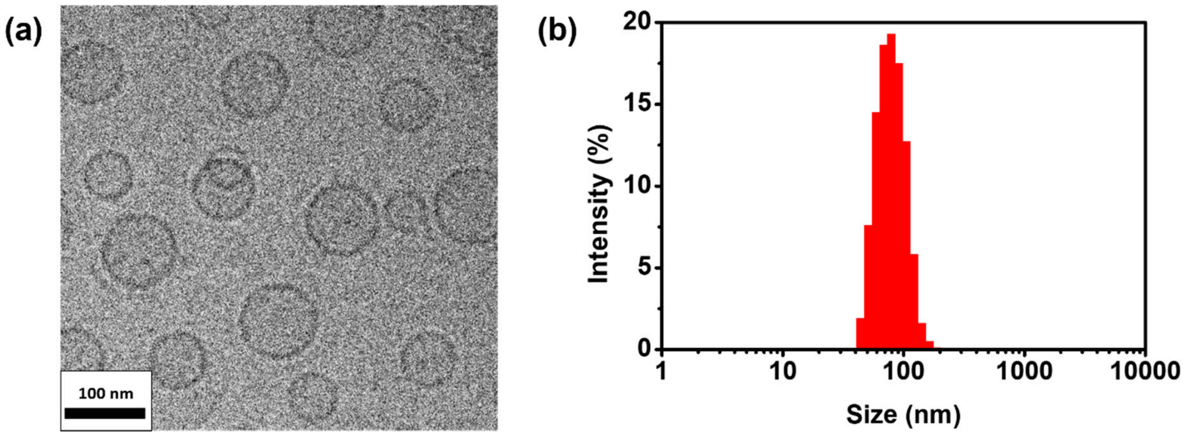

Figure S4. Characterization of liposomes (a) Cryo-TEM image of liposomes. (b) Size distribution of control liposomes using DLS.
